# Immunomodulatory Response Triggered by the Alkaloids, 3-Amino-7-Benzylbenzimidazo[3,2-*a*] Quinolinium Chloride (ABQ-48) and 3-Nitro-7-Benzylbenzimidazo [3,2-*a*] Quinolinium Chloride (NBQ-48)

**DOI:** 10.17303/jcrto.2015.103

**Published:** 2015-05-04

**Authors:** Miguel Otero, Beatriz Zayas, Eric Miranda, Christian Velez, Wigberto J. Hernandez, Luis A. Rivera, Osvaldo Cox

**Affiliations:** 1 Department of Microbiology and Medical Zoology, University of Puerto Rico Medical Sciences Campus, San Juan, Puerto Rico; 2 Universidad Metropolitana, School of Environmental Affairs, San Juan, Puerto Rico; 3 Universidad Central del Caribe, School of Medicine, Department of Microbiology and Immunology, Bayamon, Puerto Rico; 4 Department of Chemistry, University of Puerto Rico at Mayaguez, Mayaguez, Puerto Rico

**Keywords:** Anti cancer, Immune modulation, BQS, Alkaloids, Hypoxia

## Abstract

ABQ-48 (3-amino-7-benzylbenzimidazo[3,2-*a*]quinolinium chloride) and NBQ-48 (3-nitro-7-benzylbenzimidaw[3,2-*a*] quinolinium chloride) are un-natural alkaloids containing a planar heteroaromatic systems characterized by quaternized nitrogen fused to benzothiazole nucleus. Both compounds are structurally related to naturally occurring substances such as elliptine (from Ochrosia), and berberine (from Berberis). Previous in vitro studies have shown these agents to control tumor-cell proliferation indicating that both BQS are active but especially ABQ-48 at a 1 OuM dose with over 80% control of the proliferation of multiple cancer cell lines from various etiologies including colon, melanoma, CNS and ovarian cells. Mechanism of action studies have also been conducted however this is the first approach to evaluate immune modulatory activity of these novel BQS. Immune-based therapy is an increasing field in which scientists identify how the immunomodulatory activity of known and newly discovered compounds elicits an immune response that could be used against diseases. In this study, our main objective was to apply an in vitro model to show the immunomodulatory effects of ABQ-48 and NBQ-48 by analyzing the cytokine profile resulting after extracted murine spleen cells were treated with both BQS using a fluorescence-based multiplex ELISA approach. Screened cytokines included: G-CSF, GM-CSF, IL-1a, IL-2, IL-3, IL-5, IL-6, IL-7, IL-10, IL-12p70, IL-13, IL-15, IL-17, IL-21, IL-23, IFN-γ, and TNF-α. Our study results show ABQ 48 and NBQ-48 to stimulate the release of G-CSF, IL-2, IL-6, and, IFN-γ when mouse splenocytes are incubated with serial dilutions of these agents. Our finding opens new possibilities of potentially using ABQ-48 and NBQ-48 as immunomodulatory agents; with intend to activate the immune system such as the production of neutrophils against cancer or reducing chemotherapy side effects.

## Introduction

Immune based cancer therapy has become an exciting field for the discovery of novel treatments and new uses for known active substances [1-3]. Some efforts have been directed at using chemically described compounds to elicit a particular immune response such as interleukin modulation (4]. Other approaches have begun utilizing the immune response as a direct effector against cancer, by modifications to the immune response [5,6], or by therapy with cellular preparations [7].

Some groups have used small molecules as adjuvants in immune response therapy; among these the imidazoquinoline family has demonstrated an increase in cytokine production (8]. Another example from this family is imiquimod; this compound has been successfully used against squamous cell carcinoma causing apoptosis in a dose-dependent manner causing activation of the intrinsic apoptotic pathway [9]. In this study we report the immunomodulatory responses of two unnatural alkaloids 3-amino-7-benzylbenzimidaw[3,2-*a*] quinolinium chloride (ABQ-48: NSC D-763307) and 3-nitro-7-benzylbenzimidazo[3,2-*a*]quinolinium chloride (NBQ-48: NSC D -763303). The included NSC D number relates to the NCI designation as presented in Cox et al. [10].

**Figure 1:**
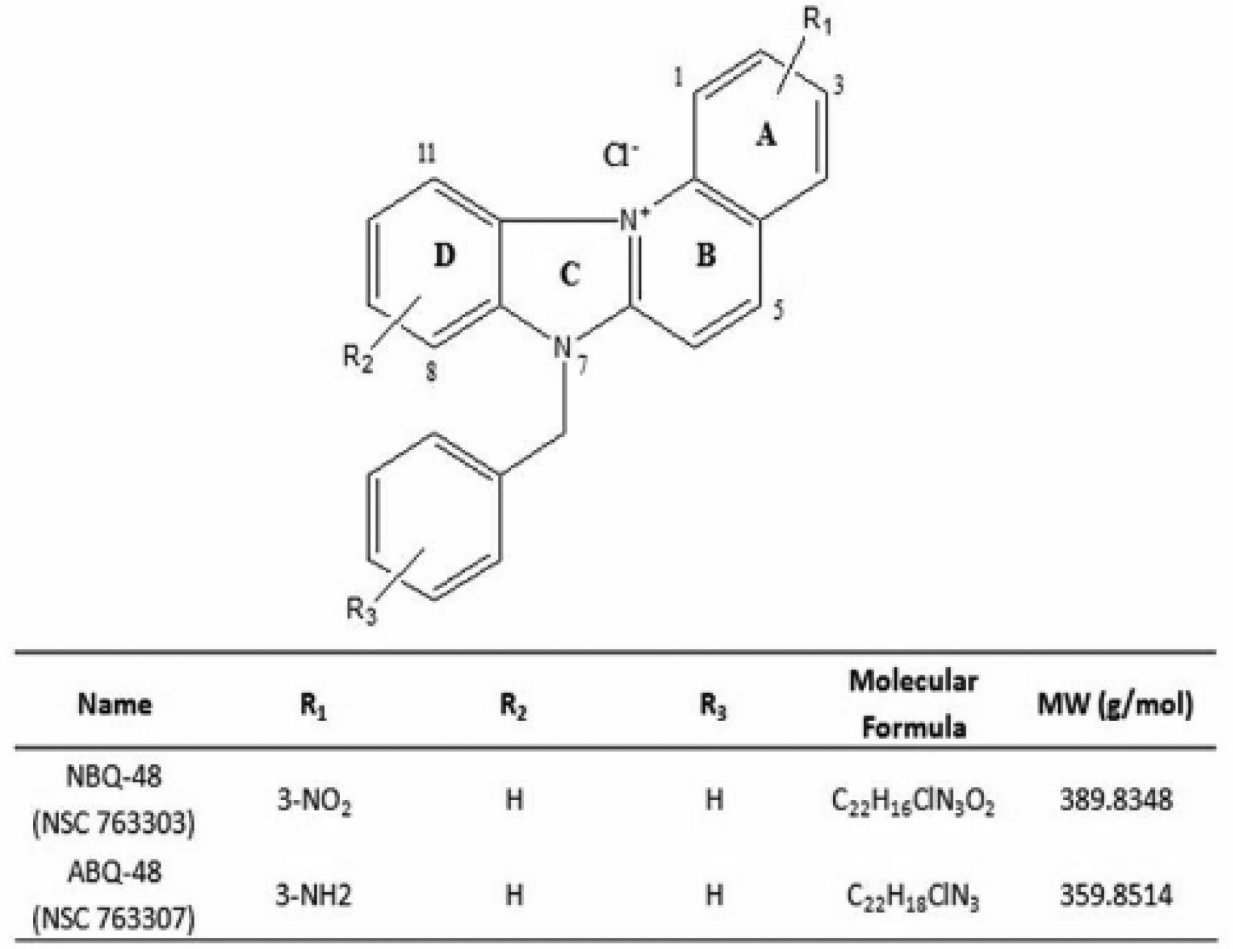
General structure of 3-amino-7-benzylbenzimidazo[3,2-*a*]quinolinium chloride (ABQ-48: NSC D-763307) and 3-nitro-7-benzylbenzimidazo[3,2-*a*]quinolinium chloride (NBQ-48: NSC D -763303)

These compounds have a planar heteroaromatic system characterized by a quaternized nitrogen and a fused benzimidazole (Figure 1). Both compounds are structurally related to naturally occurring substances such as ellipticine (from Ochrosia) and berberine (from Berberis). Ellipticine has been studied since the 1959 and has shown a variety of biological activities such as DNA interaction, anti-cancer activity and mitochondrial uptake [11]. Berberine has shown similar activities including anti cancer activity through mitochondria interactions [12]. Our previous work on other compounds from this family of benzazolo[3,2-*a*]quinolinium salt (BQS) compounds has demonstrated diverse biological activities such as growth inhibition; apoptosis induction, and DNA adduct formation in cancer types such as epidermoid carcinoma [13-15].

In this report, we use an in vitro model to show the immunomodulatory effects of ABQ-48 and NBQ-48. Specifically, we show these compounds to stimulate cytokine release when mouse splenocytes are incubated with serial dilutions of these agents. Our data shows the expression of G-CSF, IL-2, IL-6, and, IFN-γ to be higher after in vitro stimulation with ABQ 48 or NBQ-48, compared to non-stimulated cultures. Additional experiments will be performed to study how the immunomodulatory activity of ABQ-48 and NBQ-48 could be used as a resource in the immune activation against cancer.

## Materials and Methods

### Chemicals

3-amino-7-benzylbenzimidazo [3,2-*a]* quinolinium chloride (ABQ-48: NSC D-763307) and 3 nitro-7-benzylbenzimidazo [3,2-*a*] quinolinium chloride (NBQ-48: NSC D -763303) were prepared as described by Cox et al. [10].

### Mice

Female 4-6 week-old BALB/c mice were purchased from Charles River (Wilmington, MA, USA). Care of the animals was in accordance with the guidelines from The National Institutes of Health (Bethesda, MD, USA), and the University of Puerto Rico Institutional Care and Use Committee (IACUC).

### Preparation of mouse lymphocytes

Aseptically amputated spleens from anesthetized mice were pressed through a 70 μm nylon cell strainer (BD Falcon, Franklin Lakes, NY, USA) using a syringe plunger, to produce a single-cell splenocyte suspension. The resulting cells were incubated in ACK lysis buffer (Gibco, Grand Island, NY, USA), and washed in 15 mL of RPMI media supplemented with 10% PBS serum and Pen/Strep. Mice were humanely euthanized by cervical dislocation.

### Cell culture conditions

Murine splenocytes isolated from humanely euthanized mice were counted and their viability calculated using a Nexelom Biosciences Cellometer Auto T4 cell counter (Lawrence, MA, USA). Splenocytes were incubated at 2×10^6^ cells/mL in a flat-bottom 96 well plate in 100 μL of RPMI media supplemented with 10% PBS serum and Pen/Strep, in a humidified incubator at 37°C and 5% C02.

### Drug treatment

Splenocytes were incubated in twofold dilutions ranging from 5 μg/mL to 0.3 μg/mL of the BQS under study for a final volume of 200 μL. Concanavalin A (Con A, Sigma, St. Louis, MO, USA) and culture media were used as positive and negative controls, respectively. Plates were incubated in a humidified incubator at 37°C in a 5% C02 atmosphere for five days, when the plate was centrifuged, supernatants were removed and stored at -80°C until testing.

### Evaluation of Cytokine Profile

The cytokine profile resulting after murine spleen cells were treated with BQS was analyzed using a fluorescence-based multiplex ELISA microarray chip, following the protocol as indicated by the manufacturer (RayBiotech, Norcross, GA, USA). Screened cytokines included: G-CSF, GM-CSF, IL-1a, IL-2, IL-3, IL-5, IL-6, IL-7, IL-10, IL-12p70, IL-13, IL-15, IL-17, IL-21, IL-23, IFN-γ, and TNF-α.

### Statistical analysis

Cytokine-profile determination shows data from experiments that were repeated in triplicates. The immunomodulatory activities produced from each cytokine are presented as the mean ± standard error of the mean (SEM). The statistical significance of differences among cytokines was determined by One-way ANOVA, followed by the Tukey's test, using the GraphPad Prism statistical software (La Jolla, CA, USA). A p value of less than 0.05 was considered significant.

## Results

### Immuno-modulatory profile of ABQ-48 and NBQ-48

**Figure 2:**
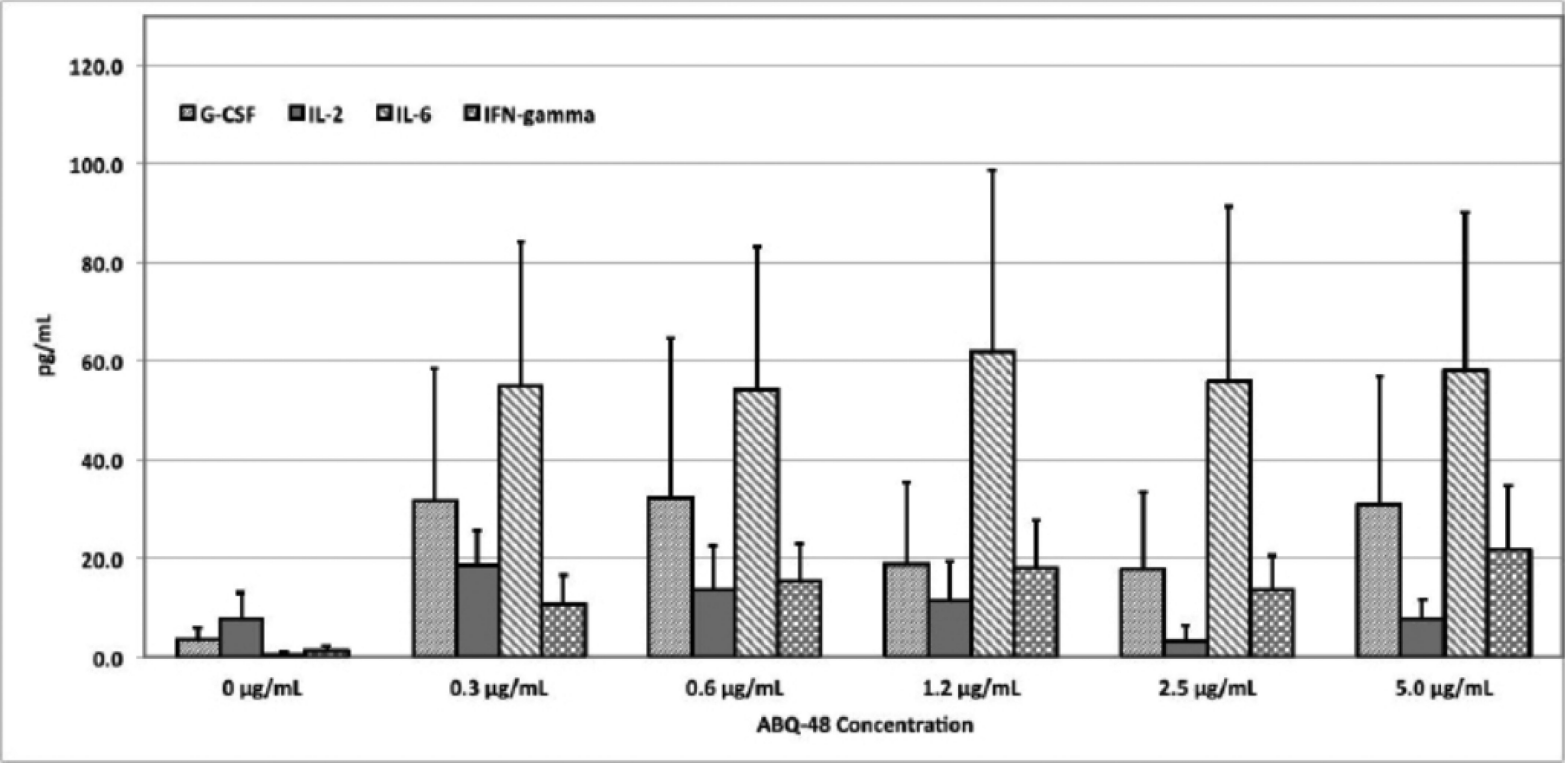
Production of G-CSF, IL-2, IL-6, and IFN-γ in culture supernatants from ABQ 48-treated lymphocyte cultures Murine splenic lymphocytes were isolated and incubated with twofold serial dilutions of ABQ-48 (5-0.3 ug/mL) for 5 days. Culture supernatants were collected and the cytokine concentrations were determined by protein microarray analysis. Supernatants from Concanavalin A-treated lymphocytes, and media alone were used as positive and negative controls, respectively. Results were expressed as mean concentrations ± SEM of three independent experiments.

**Figure 3:**
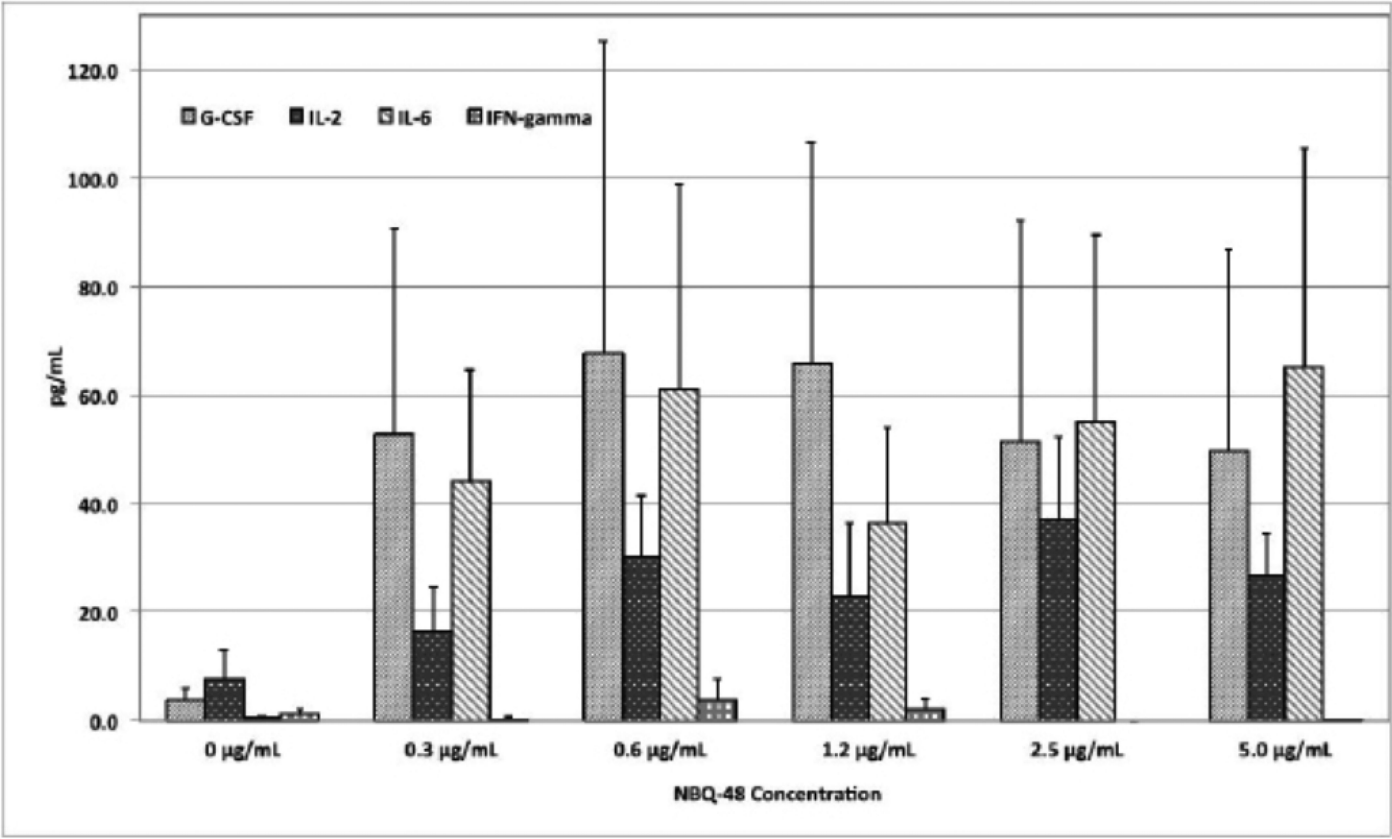
Production of G-CSF, IL-2, IL-6, and IFN-γ in culture supernatants from NBQ 48-treated lymphocyte cultures Murine splenic lymphocytes were isolated and incubated with twofold serial dilutions of NBQ-48 (5-0.3 μg/mL) for 5 days. Culture supernatants were collected and the cytokine concentrations were determined by protein microarray analysis. Supernatants from Concanavalin A-treated lymphocytes, and media alone were used as positive and negative controls, respectively. Results were expressed as mean concentrations ± SEM of three independent experiments.

The following cytokines were analyzed in this experiment: G-CSF, GM-CSF, IL-1a, IL-2, IL-3, IL-5, IL-6, IL-7, IL-10, IL-12p70, IL-13, IL-15, IL-17, IL-21, IL-23, IFN-γ, and TNF-α. Expression of G-CSF, IL-2, IL-6, , and, IFN-γ was higher after in vitro stimulation with ABQ 48 (Figure 2) or NBQ-48 (Figure 3) compared to non-stimulated cells. Interestingly, culture conditions used for ABQ-48 and NBQ-48 stimulation of mouse lymphocytes show a pro inflammatory cytokine profile. These cytokines are known to have a role in the modulation of immune responses.

Specifically, IL-6 was the highest cytokine released in culture supernatants of ABQ 48 stimulated murine lymphocytes (Figure 2), while both, IL-6 and G-CSF, were the highest after NBQ-48-mediated stimulation (Figure 3). The titers of IL-6 are constantly high after splenocyte activation using both compounds, showing no dose-response correlation to the amount of either alkaloids were used. Under these stimulation conditions ABQ-48 induced an average of 57.02±1.40 pg/mL of IL-6, while the average induced by NBQ-48 was 52.35±5.36 pg/mL. NBQ-48 was able to induce higher amounts of G-CSF as compared to ABQ 48. Specifically, NBQ-48 induced an average of 57.46±3.86 pg/mL of G CSF, while ABQ-48 induced 26.25±3.29 pg/mL of that cytokine.

As stated before, no dose-response correlation was observed in the expression of IL-6 at the concentration ranges of ABQ-48 and NBQ-48 used for stimulation, and in the expression of G-CSF among the tested concentration range for NBQ-48. Other experiments designed to test additional concentration ranges might be necessary in order to identify the linear region for the NBA-48- and ABQ-48-mediated release of these cytokines. On the other hand, Figure 2 shows that ABQ-48 induced a positive dose-response trend in the production of IFN-γ. In this case, ABQ-48-mediated release of IFN-γ ranged from 10.6 to 21.7 pg/mL. Interestingly, a negative trend in the release of IL-2 was observed when murine splenocytes were stimulated with ABQ-48, ranging from 18.5 to 3.3 pg/mL. Moreover, NBQ-48 induced higher release of IL-2 on almost all experimental conditions compared to ABQ-48. However, the NBQ-48-mediated release of IFN-γ was low under these conditions.

Among all tested conditions, the highest concentrations of the expressed cytokines resulted when ABQ-48 was used to stimulate the splenocytes with G-CSF, IL-2, IL-6, and IFN-γ levels of 32.3, 18.5, 61.9, and 21.7 pg/mL respectively. These were produced when splenocytes were stimulated with 0.6, 0.3, 1.2, and 5 μg/mL of ABQ 48, respectively (Table 1). Regarding NBQ-48, the highest concentrations of G-CSF, IL 2, IL 6, and IFN-γ were 67.6, 37.2, 65.2, and 3.8 pg/mL, produced when stimulating with 0.6, 2.5, 5.0, and 0.6 μg/mL of NBQ-48, respectively (Table 2).

These data reveals that besides the strong in vitro anti-neoplastic activity already reported for ABQ-48 and NBQ-48, these agents also have the capacity to induce a pro-inflammatory cytokine profile. Additional experiments should be performed to explore how the combination of these properties could be used to test ABQ-48 and NBQ-48 as effective anti tumor agents when combined with a relevant cancer biomarker.

## Discussion and Conclusions

The proven capacity of ABQ-48 and NBQ-48 controlling tumor-cell proliferation establishes these agents as promising antineoplastic candidates with potential clinical implications. Several antineoplastic agents used in cancer therapies are known to induce adverse side effects like severe neutropenia [16], thrombocytopenia, nausea [17,18], and susceptibility to infections [19]. These undesired effects have a negative impact in the quality of life in patients. For many patients, not only the cancer, but also the therapy itself undermines their daily activities. In this regard, several anticancer chemotherapies are combined with G-CSF (Filgrastim, commercially traded as Neupogen*, or Pegfilgrastim commercially traded as Neulasta*) to stimulate proliferation and differentiation of granulocytes [20]. This combination is effectively used to enhance the quality of life of patients suffering from cancer [21,22] by restoring the levels of neutrophils in blood [23-25].

Cytotoxic studies of ABQ-48 and NBQ-48 show these compounds to control tumor proliferation, which until now have been the major research focus of these two novel generated compounds and other members of their family. However, it is of great interest to further evaluate the observed cytokine stimulating activities of these compounds. The results of this study clearly demonstrate these BQS to stimulate the secretion of G-CSF, an agent that, as stated before, is used in combination with anticancer chemotherapy to activate the production of neutrophils [23,26]. This combination therapy has been shown to enhance the quality of life in cancer patients [23-25]. This study shows ABQ-48 and NBQ-48 to effectively induce both effects: control of tumor growth and immune modulation to produce G-CSF, among other cytokines. Therefore, ABQ-48 and NBQ-48 could induce tumor-proliferation control with minimal side effects after stimulating proliferation and differentiation of granulocytes [20] and at some degree restoring the levels of neutrophils in blood.

Moreover, in this report, NBQ-48-mediated stimulation of murine splenocytes is shown to produce G-CSF, IL-2 and IL-6, while ABQ-48 also mediates the release of IFN γ. These are pro-inflammatory cytokines with a relevant role in the activation of immune responses. This finding opens the new possibility of using ABQ-48 and NBQ-48 as immunomodulatory agents, with intend to activate the immune system against cancer. However, this information will also be used in the rational design of new antineoplastic agents capable of modulating the immune response. These data set the bases for the development of novel compounds with immunomodulatory capabilities. In this regard, projects by several groups have focused on the development of immunomodulators with intent to induce an immune response against cancer [27,28].

Our exciting ex vivo data encourages us to test the immunomodulatory effects of these agents in an in vivo mouse model. We believe that it is crucial to test the effects of these agents in a model where the whole pharmacokinetics, drug metabolism and toxicological factors influence the immunomodulatory responses. First, it is necessary to determine the impact that these agents have in the in vivo proliferation of granulocytes using a mouse model. This will tell us if these agents could be able to restore that important compartment of the immune system, as it is depleted during anticancer therapies.

It is important however to mention that existing cancer research literature, provide some arguments on the effects of certain pro-inflammatory cytokines and cancer progression. Other correlations however, such as antitumor effects or the prevention of chemotherapy secondary effects have also been associated with the over expression of certain cytokines, especially G-CSF [29,30]. For example, Maeda H. et al, 1994, reported the antitumor effects of the combination of G-CSF and TNF on a xenograft line of human medulloblastoma [29]. Also the combination of G-CSF a key regulator of neutrophil production has been integrated to chemotherapy protocols especially for certain types of Non Hodgkin's Lymphoma[30]. Keeping this in mind and based on the obtained results, it would be worth to test the adjuvant potential of ABQ-48 and/or NBQ-48. In this regard, the development of a vaccine formulation consisting of these tested BQS combined with a cancer-specific biomarker could be expected to induce a protective antigen-specific immune response against a selected tumor. The tumor controlling characteristics of this formulation, together with the generation of G CSF, and the induced immunostimulatory profile of these compounds, makes these agents strong candidates to be tested as safe immune-modulators in a pre-clinical cancer regimen.

## Figures and Tables

**Table 1:** Maximal concentrations of cytokines produced in culture supernatants from AEQ-48-treated lymphocyte cultures.

ABQ-48, μg/mL	Cytokine	Cytokine concentration, pg/mL±SEM
0.6	G-CSF	32.3±22.8
0.3	IL-2	18.5±7.2
1.2	IL-6	61.9±20.4
5.0	TFN-γ	21.7±12.8

Murine splenic lymphocytes were isolated and incubated with twofold serial dilutions of ABQ-48 (5-0.3 μg/mL) for 5 days. Culture supernatants were collected and the cytokine concentrations were determined by protein microarray analysis. Supernatants from Concanavalin A-treated lymphocytes, and media alone were used as positive and negative controls, respectively. Results were expressed as mean concentrations ± SEM of three independent experiments.

**Table 2:** Maximal concentrations of cytokines produced in culture supematants from NEQ-48-treated lymphocyte cultures.

NBQ-48, μg/mL	Cytokine	Cytokine concentration; pg/mL±SEM
0.6	G-CSF	67.6±57.5
2.5	IL-2	37.2±15.1
5.0	IL-6	65.2±40.2
0.6	IFN-γ	3.8±3.8

Murine splenic lymphocytes were isolated and incubated with twofold serial dilutions of NBQ-48 (5-0.3 μg/mL) for 5 days. Culture supernatants were collected and the cytokine concentrations were determined ay protein microarray analysis. Supernatants from Concanavalin A-treated lymphocytes, and media alone were used as positive and negative controls, respectively. Results were expressed as mean concentrations ± SEM of three independent experiments.
